# Severe sepsis: Low expression of the renin-angiotensin system is associated with poor prognosis

**DOI:** 10.3892/etm.2014.1566

**Published:** 2014-02-20

**Authors:** WEI ZHANG, XIAOWEI CHEN, LING HUANG, NING LU, LEI ZHOU, GUOJIE WU, YUGUO CHEN

**Affiliations:** 1 Department of Emergency, Qilu Hospital of Shandong University, Jinan, Shandong 250012, P.R. China; 2 Intensive Care Unit, Yantaishan Hospital, Yantai, Shandong 264001, P.R. China

**Keywords:** severe sepsis, angiotensin II, angiotensin-converting enzyme, mortality, prognosis

## Abstract

Severe sepsis has a high fatality rate, but no clinical indices for prognosis have been established. In recent years, the renin-angiotensin system (RAS) has received considerable attention. However, clinical data on RAS are inconsistent. Therefore, the aim of the present study was to assess the significance of RAS in the prognosis of sepsis. Blood samples were collected from patients, who met the diagnostic criteria of severe sepsis, on day 1 (D1) and 3 (D3). For each sample, the levels of angiotensin II (AngII), angiotensin-converting enzyme (ACE) and additional indices were measured. Patients were monitored for 28 days. On the D1 of inclusion, the average Acute Physiology and Chronic Health Evaluation II (APACHE II) score was 22.2 and the Sepsis-related Organ Failure Assessment (SOFA) score was 6.1. Logistic regression analysis revealed that mortality-associated variables included the APACHE II score on D1, the SOFA score on D1, high lactic acid levels on D3 and low AngII and ACE levels on D1 and D3. AngII levels (<86.1 ng/ml) on D1 had a sensitivity of 88.2% and specificity of 77.3% for predicting mortality. ACE levels (<39.2 ng/ml) on D1 had a sensitivity of 88.2% and specificity of 72.7% for predicting mortality. These two indices were better than the APACHE II and SOFA scores. Therefore, low expression levels of AngII and ACE are valuable in predicting the mortality of patients with severe sepsis.

## Introduction

Sepsis-associated mortality rates have not decreased despite powerful technical support and development of advanced treatments. Severe sepsis remains the most common cause of mortality in intensive care units (ICUs) (1). The mortality rate of severe sepsis is 25–30%, whereas the mortality rate of septic shock at the most severe phase of sepsis is as high as 40–70% (2). Therefore, identifying septic patients that may have the worst outcomes is crucial. Certain clinical indices, including multiple organ dysfunction and high disease risk score, have been shown to be associated with clinical prognosis (3); however, the application of these indices is difficult. Therefore, relevant laboratory variables are required for sepsis prognosis. In recent years, the renin-angiotensin system (RAS) has received increasing attention in the field of sepsis, but the clinical research results on RAS are inconsistent. Previous studies have shown that RAS may be activated in patients with severe sepsis (4), which may subsequently result in ischemic reperfusion injury (5) or energy metabolic abnormality (6). RAS antagonists may also be applied for the treatment of sepsis (7). In addition, a previous study demonstrated that following the occurrence of sepsis, the expression of the angiotensin receptor (ATR) was downregulated (8). Exogenous angiotensin II (AngII) may be used to enhance urine volume and the creatinine clearance rate (9), as well as treat specific patients with septic shock who are insensitive to norepinephrine (10). Therefore, the aim of the present study was to monitor the dynamic changes of RAS in patients with severe sepsis. The significance of RAS in the prognosis of sepsis was evaluated by comparing the RAS levels in patients with various clinical outcomes.

## Subjects and methods

### Subjects

Patients with severe sepsis (including septic shock) were included in this study. The individuals were admitted to the ICU of Yantai Mountain Hospital (Yantai, China) between January 2011 and December 2011. All the patients satisfied the diagnostic criteria of the Conference of Washington in 1992 (11). Sepsis was diagnosed by the presence of systemic inflammatory reaction syndrome and bacterial infection. Severe sepsis includes complications such as organ dysfunction or tissue hypoperfusion. Septic shock is a type of hypotension in which fluid resuscitation is ineffective. Patients suffering from septic shock have a systolic blood pressure (SBP) of <90 mmHg (1 mmHg = 0.133 kPa) or a mean arterial pressure of <70 mmHg. Patients may also exhibit an SBP decrease of >40 mmHg or reduction of 2 standard deviation based on age that is >2 if no other evident causes of hypoperfusion are observed. All the patients in this study were observed within 24 h after the onset of severe sepsis or septic shock. The study was conducted in accordance with the Declaration of Helsinki and with approval from the Ethics Committee of Qilu Hospital of Shandong University (Yantai, China). Written informed consent was obtained from all participants.

### Exclusion criteria

Patients under the following conditions were excluded from the study: Patients with chronic renal failure that had received hemodialysis or ultrafiltration; patients with acute renal failure that had received urgent hemodialysis; patients with terminal conditions whose life expectancy was <48 h; patients who were pregnant or lactating; and patients aged <18 years.

### Collection of specimens

Venous blood samples were collected from patients, who satisfied the diagnostic criteria of severe sepsis, on day 1 (D1) and 3 (D3) after diagnosis. For each sample, the levels of AngII, angiotensin-converting enzyme (ACE), AngII type 1 receptor (AT1R) antagonist and AngII type 2 receptor (AT2R) antagonist were measured, as well as the levels of pro-brain natriuretic peptide (pro-BNP), troponin T (TNT), C-reactive protein (CRP) and lactate. Acute Physiology and Chronic Health Evaluation II (APACHE II) and Sepsis-related Organ Failure Assessment (SOFA) scores were calculated within the first 24 h. Patient medical and drug usage history, specifically ACE inhibitor (ACEI) or AngII receptor antagonist (ARB), were recorded. Observation lasted for 28 days. Follow-up was conducted via telephone calls for patients who had left the ICU or hospital prior to day 28.

### Treatment principles of severe sepsis

Upon admission, the patients received crystal solution or colloid solution within the first 6 h for early recovery, based on the Surviving Sepsis Campaign Guidelines for Management of Severe Sepsis and Septic Shock in 2008 (2). Imaging examination was conducted immediately to detect potential infectious lesions. Within 1 h following definitive diagnosis of severe sepsis or septic shock, wide-spectrum antibiotics were administered. If blood pressure remained <65 mmHg following fluid resuscitation, norepinephrine or dopamine were jointly administered to stabilize circulation. If this was unsuccessful in controlling the blood pressure to an ideal level, a daily dose of 200 mg succinyl hydrocortisone was administered. For patients with acute lung injury/acute respiratory distress syndrome (ALI/ARDS), ventilation with a small tidal volume or inhibition of pause pressure was applied and the management of blood glucose was enhanced.

### Diagnostic criteria for acute kidney injury (AKI)

Diagnostic criteria for AKI according to the AKI Network (12) were as follows: Sudden loss of renal function (within 48 h), which manifests as absolute elevation of serum creatinine levels to ≥0.3 mg/dl (≥26.4 mmol/l), an increase of serum creatinine levels from the baseline of ≥50% or decreased urine volume to <0.5 ml/kg/h lasting >6 h.

### Diagnostic criteria for ALI/ARDS

Diagnostic criteria for ALI/ARDS, as recommended by the American Thoracic Society and European Society of Intensive Care Medicine in 1992 (13), were as follows: i) Acute onset; ii) Diagnosis of ALI if the arterial blood oxygen partial pressure/content of oxygen inhalation (PaO_2_/FiO_2_) is ≤300 mmHg (without considering if the positive end expiratory pressure was used or not) and diagnosis of ARDS if PaO_2_/FiO_2_ is ≤200 mmHg; iii) X-ray chest film showing infiltrates in both lobes of the lung; and iv) pulmonary artery wedge pressure ≤2.4 kPa (18 mmHg) or no clinical evidence of high left atrial pressure.

### Testing of the specimens

The method used to detect the levels of AngII, ACE, AT1R and AT2R was as follows: 2-ml blood samples were collected and centrifuged at 1,760 × g for 10 min to separate the serum and erythrocytes. The samples were then stored in a refrigerator at −80°C. Levels of the variables were determined using an enzyme-linked immunosorbent assay (Shanghai Yuanye Biotechnology Co. Ltd., Shanghai, China). Pro-BNP and TNT kits were provided by Roche R&D Center (Shanghai, China) and the levels of pro-BNP and TNT were determined using electroluminescence in the clinical laboratory of our hospital. The levels of CRP (Beckman Coulter Inc., Miami, FL, USA) and lactate (Radiometer Medical ApS, Brønshøj, Denmark) were also determined in Yantaishan Hospital. The CRP kits were provided by Beckman Coulter Inc. The levels of CRP were determined using scattering immunonephelometry by IMMAGE-800 specific protein detection equipment (Beckman Coulter Inc.) in the clinical laboratory of Yantaishan Hospital. The lactate kits were provided by Radiometer Medical (ApS, Brønshøj, Denmark). The levels of lactate were determined by ABL520 (Radiometer Medical ApS, Brønshøj, Denmark) in the clinical laboratory of Yantaishan Hospital. The levels of pro-BNP and TNT were determined by PPE Roche automatic biochemical immunological analyzer (Mannheim, Germany).

### Statistical analysis

Statistical analysis was performed using SPSS software version 21.0 (IBM, Armonk, NY, USA). Data are expressed as the mean ± SD. Normal distributions of measuring materials for the two groups were compared using the univariate t-test. Measuring materials not within a normal distribution were converted to an exponential form and revalidated to identify whether they were in the normal distribution. If not, the rank-sum test was applied. Counting materials were compared using the χ^2^ test. Intergroup comparison was conducted using univariate analyses. In the univariate analysis, step-wise selection was used for variables with P<0.1 to build logistic regression models and to calculate the odds ratio and 95% confidence intervals for the risk factors and mortality. Receiver operating characteristic (ROC) curves were constructed with risk factors as the test variables and mortality as the state variable. The area under the curve (AUC) was calculated to evaluate the accuracy of the prognosis forecast. Models with accuracy of >0.7 were considered to be of clinical value. P<0.05 was considered to indicate a statistically significant difference. The variables with prognosis significance were analyzed to determine the critical value, sensitivity and specificity.

## Results

### General information

**Figure 1:**
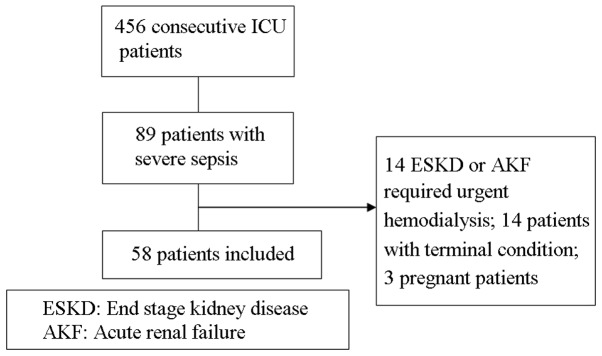
Inclusion of patients. ICU, intensive care unit.

Among the 456 continuous patients admitted to the ICU, 89 cases were diagnosed with severe sepsis (including septic shock). These 89 cases included 14 cases of end-stage kidney disease with long-term hemodialysis or acute renal failure/urgent hemodialysis or hemofiltration, 14 cases of terminal stage sepsis (6 cases in which the patient succumbed upon admission and 8 cases in which the patient succumbed within 48 h) and three pregnant females. These 31 patients were excluded from the study. Thus, a total of 58 patients were included in the study as shown in Fig. 1. The 58 patients had a mean age of 75 years and 43 patients were male. Thrombosis was the most common disease, followed by chronic obstructive pulmonary disease (COPD) and coronary heart disease. Five patients had received ACEI or ARB prior to admission. On D1 of admission, the mean APACHE II and SOFA scores were 22.2 and 6.1, respectively. The lung was the most common infection site. The majority of this group were medical patients. A total of 50 patients were admitted to the ICU due to respiratory failure and 34 patients had unstable circulation. Following admission, 49 patients required mechanical ventilation, 34 patients received pressor agents and 30 patients were administered cortical hormones. Among the 58 patients, 24 patients succumbed and 34 patients survived, resulting in a 28-day mortality rate of 41.3% (Table I).

### Comparison between the survival and mortality groups

Basic information from the survival and mortality groups was used for univariate analysis. All the patients in the mortality group presented with basic diseases, particularly thrombosis. In the survival group, the most common disease was chronic obstructive lung disease. Three patients with lung disease complicated with infection were treated, however all patients succumbed. APACHE II and SOFA scores were significantly higher in the mortality group compared with the survival group. The most common infection site was the lung in the two groups. All patients with infections in the urinary system or skin soft tissues survived. In terms of infection in multiple sites, the survival group had two cases with lung and urinary infections and one case with lung and biliary infections, whereas the mortality group had two cases with biliary and lung infections (Table I).

The disease source was not postoperative in either group and there were no intergroup differences. The major reason for admission to the ICU was respiratory failure and/or septic shock. The number of respiratory failures did not differ between the groups; however, the number of shocks was significantly larger in the mortality group compared with the survival group. The number of patients who were administered vasoactive agents and glucocorticoids was markedly larger in the mortality group compared with the survival group. With regard to complications, the number of patients with shock, AKI and ARDS was significantly larger in the mortality group compared with the survival group.

### Univariate analysis

Intergroup comparisons of laboratory variables, including the levels of AngII on D1 and D3 and ACE on D1, revealed the variable levels to be significantly higher in the survival group compared with the mortality group. However, the pro-BNP and lactic acid levels on D3 were higher in the mortality group (Table II). Variables that had a significance value of P<0.1 were included for logistic regression analysis.

### Logistic regression analysis

**Figure 2:**
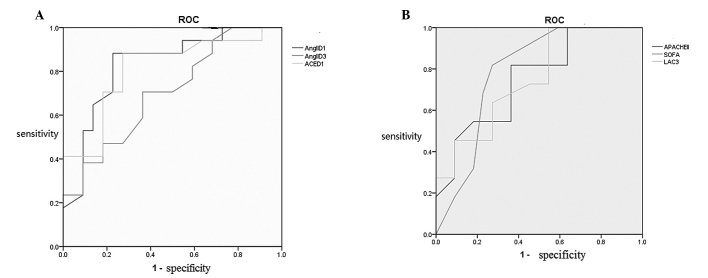
ROC curves of the mortality-associated risk factors. ROC, receiver operating characteristic; AngII, angiotensin II; ACE, angiotensin-converting enzyme; APACHE II, Acute Physiology and Chronic Health Evaluation II; SOFA, Sepsis-related Organ Failure Assessment; Lac, lactate; D, day.

Logistic regression analysis revealed that the mortality-associated variables included the APACHE II score on D1, the SOFA score on D1 and high lactic acid levels on D3, as well as low AngII levels on D1 and D3 and low ACE levels on D1 (Table III). These risk factors, determined by logistic regression analysis, were used for ROC curve analysis. APACHE II and SOFA scores on D1 and high lactic acid levels on D3 were valuable for mortality prediction. In addition, low AngII levels on D1 and D3, as well as low ACE levels on D1, may predict poor prognosis (Fig. 2). Critical values, sensitivity and specificity were calculated for the variables with an AUROC of >0.7, based on Youden’s index. The results demonstrated that AngII and ACE levels on D1 had the highest sensitivity and specificity for the prediction of mortality, followed by the SOFA score. APACHE II score showed high sensitivity but low specificity, whereas lactate levels on D3 showed high specificity but low sensitivity (Table IV).

## Discussion

In the present study, 58 cases of severe sepsis (including septic shock) were analyzed for the detection of RAS activity-associated, myocardial damage (TNT), pro-BNP, response tissue perfusion (lactate) and inflammatory (CRP) variables. Patients were medical patients with a mean age of 75 years. The major reason for admission into the ICU was due to respiratory failure or complications caused by septic shock. The lung was the most common infection site. The majority of patients in the two groups required mechanical ventilation. With regard to basic diseases, COPD was common in the survival group, but not in the mortality group. The major reason for the use of mechanical ventilation was acute exacerbations of COPD in the survival group and ARDS in the mortality group. The mean APACHE II score on D1 was 22. The 28-day mortality rate was 41%, which is consistent with the mortality rate indicated in the Guidelines for Management of Severe Sepsis and Septic Shock in 2008 (2).

Previous studies have demonstrated that a number of factors, including age, severity of basic diseases, number of injured organs/systems, disease severity score, lactic acid levels and cellular factors, affect the prognosis of severe sepsis and septic shock (14–18). Among the 58 patients in the present study, the APACHE II and SOFA scores and disease and organ/system damage severities were found to be associated with poor prognosis, which was consistent with previous studies (19,20). In addition, high lactic acid levels on D3 indicated a high mortality risk, whereas continuously high lactic acid levels on D3 following early positive treatment and recovery capacity may indicate a severe condition and high mortality risk.

The present study on RAS variables has demonstrated that relatively low expression levels of RAS are associated with poor prognosis. RAS is an important neuroendocrine system. In cases with insufficient capacity or decrease of blood pressure, circulating angiotensin I, under the action of ACE, is hydrolyzed to AngII, which is the primary active peptide in RAS. AngII functions primarily by combining with ATR to cause systemic micro-artery contraction and increase peripheral resistance and blood pressure. AngII may also enhance the release of norepinephrine from sympathetic nerve endings. Results from previous studies are controversial. One study demonstrated that sepsis is likely to result in high expression levels of RAS (4) and that RAS was involved in several developmental stages of sepsis. AngII may promote the synthesis of proinflammatory cell factors and chemokines, aggravate inflammatory reaction and increase the production of active oxygen (21). Endotoxin-treated mice showed high expression levels of RAS, which was associated with oxidative stress and endodermic dysfunction (22). A number of animal experiments have shown that RAS antagonists may be used to alleviate inflammatory reactions in septic animals and protect organ/system functions (23–25). Therefore, RAS antagonists are recommended for the treatment of sepsis (7). However, this topic remains controversial. *Escherichia coli* endotoxins may inhibit the activity of renin renal mesangial cells, resulting in low expression of RAS (26). In sepsis models, adrenal ATR is expressed in low levels, alleviating the irritation of AngII to the adrenal gland and thereby resulting in a decrease in the release of catecholamine and an induction of septic shock (27). Endotoxins can deactivate ACE and therefore decrease the levels of AngII (28). From a therapeutic perspective, Yunge and Petros used exogenous AngII to treat two children under septic shock who were insensitive to norepinephrine and the conditions improved (10). Additional studies have shown that RAS antagonists do not improve the prognosis of animals under septic shock (29,30). However, the expression levels of AngII may differ from phase to phase (31).

The results of the present study showed that the expression levels of AngII and ACE were low in the mortality group. This group exhibited complications due to septic shock, thus, vasoactive agents should be used in combination to maintain blood pressure. Therefore, patients under septic shock may react slightly to microcirculatory disturbance. Considering the lack of RAS excitation, we hypothesize that relatively low levels of AngII reduce the irritation of the adrenal cortex to release catecholamine or inhibit the ATR on the surface of adrenal gland, thereby resulting in relatively less endogenous catecholamine. Consequently, the body depends on exogenous catecholamines to maintain blood pressure. Such patients may present downregulated excitability in other systems, including the sympathetic nervous system and pituitary-adrenal axis, since the mortality group require more glucocorticoids for treatment. However, in using this method, patients are more prone to develop organ damage (high SOFA score) or have a high risk of mortality (32).

The current results are not entirely consistent with previous studies due to the following reasons. Firstly, certain patients with severe sepsis in the present study developed shock, although others did not. Secondly, differences between the survival and mortality groups were compared for the first time. However, previous studies were conducted mostly with animals and determination of variable levels was mainly performed at certain time point. Based on the current results, we hypothesize that at various levels or stages of sepsis, the expression levels of RAS may differ. Relatively low levels of RAS expression upon onset demonstrate significance for the poor prognosis of sepsis. However, the sample size in the present study was small; therefore, future studies with larger sample sizes are required for further analyses to support the conclusions.

## Figures and Tables

**Table I:** Clinical data of patients with severe sepsis.

Item	Survival group (n=34)	Mortality group (n=24)	Total (n=58)	P-value
Male, n (%)	26 (76.4)	17 (70.8)	43 (74.1)	0.629
Age, years	69.2±17.5	74.6±10.8	71.5±15.2	0.157
No comorbidity	4 (11.8)	0	4 (6.9)	0.082
Comorbidity, n (%)				
Cerebral infarction	12 (35.3)	9 (37.5)	21 (36.2)	0.863
COPD	11 (32.4)	3 (12.5)	14 (24.1)	0.082
CHD	6 (17.6)	8 (33.3)	14 (24.1)	0.169
HTN	4 (11.8)	5 (20.8)	9 (15.5)	0.347
ACEI/ARB	3 (8.8)	2 (8.3)	5 (8.6)	0.948
Diabetes mellitus	4 (11.8)	3 (12.5)	7 (12.1)	0.933
Pneumoconiosis	0	3 (12.5)	3 (5.2)	0.034
APACHE II	19.8±6.3	25.5±6.0	22.2±6.7	0.001
SOFA	5.1±2.2	7.3±1.7	6.1±2.3	<0.001
Source of infection, n (%)				
Pneumonia	27 (79.4)	22 (91.7)	49 (84.5)	0.204
Urosepsis	4 (11.8)	0	4 (6.9)	0.082
Biliary infection	1 (2.9)	2 (8.3)	3 (5.2)	0.361
Soft tissue infection	2 (5.8)	0	2 (3.4)	0.227
Multiple foci	3 (8.8)	2 (8.3)	5 (8.6)	0.948
Treated type, n (%)				
Elective surgery	1 (2.9)	0	1 (1.7)	0.397
Emergency surgery	5 (14.7)	2 (8.3)	7 (12.1)	0.463
Medical	28 (82.4)	22 (91.7)	50 (86.2)	0.311
Cause of ICU admission, n (%)				
Respiratory failure	29 (85.3)	21 (87.5)	50 (86.2)	0.810
Shock	14 (41.2)	20 (83.3)	34 (58.6)	0.001
Treatment in ICU, n (%)				
Mechanical ventilation	25 (73.5)	24 (100)	49 (84.5)	0.032
Vasopressor agents	14 (41.2)	20 (83.3)	34 (58.6)	0.001
Use of glucocorticoids	12 (35.3)	18 (75)	30 (51.7)	0.003
Complication, n (%)				
Shock	14 (41.2)	20 (83.3)	34 (58.6)	0.001
AKI	11 (32.4)	17 (70.8)	28 (48.3)	0.004
ALI/ARDS	12 (35.3)	18 (75)	30 (51.7)	0.003

COPD, chronic obstructive pulmonary disease; CHD, coronary heart disease; HTN, hypertension; ACEI, angiotensin-converting enzyme inhibitors; ARB, angiotensin receptor antagonist; APACHE II, Acute Physiology and Chronic Health Evaluation II; SOFA, Sepsis-related Organ Failure Assessment; ICU, intensive care unit; AKI, acute kidney injury; ALI/ARDS, acute lung injury/acute respiratory distress syndrome.

**Table II:** Laboratory parameters.

Item	Survival group (n=34)	Mortality group (n=24)	Total (n=58)	P-value
AngII, pg/ml				
D1	91.39±6.04	83.29±6.18	88.04±7.26	<0.001
D3	72.83±7.53	66.23±6.81	70.10±7.89	<0.001
ACE, U/l				
D1	41.45±1.95	38.97±1.29	40.29±2.02	<0.001
D3	34.30±1.46	33.69±1.62	34.05±1.54	0.160
AT1R, ng/ml				
D1	3.93±0.57	3.67±0.47	3.82±0.54	0.079
D3	3.76±0.52	3.67±0.37	3.72±0.46	0.490
AR2R, ng/ml				
D1	4.57±0.72	4.20±0.64	4.43±0.70	0.060
D3	4.37±0.72	4.26±0.46	4.32±0.62	0.537
pro-BNP, pg/ml				
D1	4,246.01±9,475.85	7,378.27±9,342.36	5,691.66±9,454.33	0.237
D3	2,712.78±4,508.00	10,106.44±11,495.76	6,215.04±9,227.76	0.018
TNT, ng/ml				
D1	0.11±0.23	0.11±0.10	0.11±0.18	0.924
D3	0.26±0.49	0.20±0.18	0.22±0.35	0.623
CRP, mg/dl				
D1	95.69±52.28	81.84±58.97	89.48±55.16	0.390
D3	71.79±51.39	80.74±46.14	76.05±48.57	0.558
Lac, mmol/l				
D1	1.82±2.67	2.52±1.84	2.13±2.34	0.283
D3	1.48±0.69	3.90±4.20	2.69±3.21	0.014

AngII, angiotensin II; ACE, angiotensin-converting enzyme; ATIR, angiotensin type 1 receptor; AT2R, angiotensin type 2 receptor; pro-BNP, pro-brain natriuretic peptide; TNT, troponin T; CRP, C-reative protein; Lac, lactate; D, day.

**Table III:** Multifactor logistic regression analysis associated with mortality from severe sepsis.

Item	B	SE	OR	95% CI	P-value
AngII (D1)	−0.219	0.061	0.803	0.712–0.905	0.001
AngII (D3)	−0.132	0.045	0.877	0.802–0.958	0.004
ACE (D1)	−0.804	0.236	0.448	0.282–0.711	0.001
Lac (D3)	1.231	0.534	3.426	1.203–9.757	0.021
SOFA (D1)	0.538	0.165	1.713	1.24–2.367	0.001
APACHE II (D1)	0.153	0.054	1.166	1.050–1.295	0.004

AngII, angiotensin II; ACE, angiotensin-converting enzyme; Lac, lactate; SOFA, Sepsis-related Organ Failure Assessment; APACHE II, Acute Physiology and Chronic Health Evaluation II; D, day; SE, standard error; OR, odds ratio; CI, confidence interval.

**Table IV:** Critical values, sensitivity and specificity of the mortality-associated variables.

Item	Critical values	Sensitivity (%)	Specificity (%)
AngII (D1)	86.1	88.2	77.3
ACE (D1)	39.2	88.2	72.7
SOFA (D1)	5.5	81.8	72.7
AngII (D3)	67.9	70.6	63.6
Lac (D3)	2.3	45.5	91.1
APACHE II (D1)	19	81.8	36.4

AngII, angiotensin II; ACE, angiotensin-converting enzyme; SOFA, Sepsis-related Organ Failure Assessment; Lac, lactate; APACHE II, Acute Physiology and Chronic Health Evaluation II; D, day.
